# Supplementary material for: Primary health clinic toilet/bathroom surface swab sampling can indicate community profile of sexually transmitted infections
Source: PeerJ. 2017 Jun 22;5:e3487. doi: 10.7717/peerj.3487 (PMC5483037; doi:10.7717/peerj.3487)
Supplement: Supplemental Information 1 [file peerj-05-3487-s001.docx]

**Complete Numeric Data**

As described in the “Methods”, STI notifications data were derived from the Northern Territory Sexual Health and Blood Borne Viruses Unit Surveillance Updates, Volume 13, parts 1 and 2: <http://digitallibrary.health.nt.gov.au/prodjspui/bitstream/10137/237/101/Surveillance%20Update%20Vol%2013%20no%201%20January-March%20%26%20April-June%202012%20.pdf>

<http://digitallibrary.health.nt.gov.au/prodjspui/bitstream/10137/237/102/Surveillance%20Update%20Vol%2013%20no%202%20July-September%20%20%26%20October-December%202012.pdf>

The swabs data were derved from Table 1 of “Andersson et al, , Journal of the Pediatric Infectious Diseases Society 2014;**3**(3):189-96.”. As described in the “Methods”, normalisation was performed to correct for instances wwhere fewer than the default number of 140 tests were performed for each STI agent/clinic combination. The modified version of the table is shown below with the normalised figures added. For the two instances where 139 rather than 140 tests were performed (remote Indigenous clinic D), no normalised figures are shown because the change was too small to affect subsequent calculations, given the number of significant figures used.

|  | **SARC** | | **Sex. Health** | | **Regional** | | **Remote Indigenous** | | | |
| --- | --- | --- | --- | --- | --- | --- | --- | --- | --- | --- |
|  | **A** | **B** | **A** | **B** | **A** | **B** | **A** | **B** | **C** | **D** |
| ***C. trachomatis*** | **0**  (0 / 139) | **0**  (0 / 140) | **0**  (0 / 140) | **0.7**  (1 / 140) | **5.0**  (7 / 140) | **0**  (0 / 140) | **3.6**  (5 / 140) | **6.4**  (9 / 140) | **7.1**  (10 / 140) | **9.4**  (13 / 139) |
| ***N. gonorrhoeae*** | **0**  (0 / 140) | **0**  (0 / 140) | **6.4**  (9 / 140) | **5.0**  (7 / 140) | **0**  (0 / 140) | **0**  (0 / 140) | **0**  (0 / 140) | **2.1**  (3 / 140) | **25.0**  (35 / 140) | **36.7**  (51 / 139) |
| ***T. vaginalis*** | **0**  (0 / 140) | **0**  (0 / 140) | **0.7**  (1 / 140) | **0.7**  (1 / 140) | **7.1**  (10 / 140) | **14.3**  (20 / 140) | **26.1**  (31 / 119) | **44.2**  (53 / 120) | **37.9**  (53 / 140) | **32.1**  (45 / 140) |
| ***T. vaginalis***  **Normalised** |  |  |  |  |  |  | **36/140** | **62/140** |  |  |
| **Positive swabs**  (any STI agent) | **0**  (0 / 140) | **0**  (0 / 140) | **7.1**  (10 / 140) | **6.4**  (9 / 140) | **10**  (14 / 140) | **14.3**  (20 / 140) | **24.3**  (34 / 140) | **40.7**  (57 / 140) | **50.7**  (71 / 140) | **57.1**  (80 / 140) |
| **Clinic category**  **total** | **0** (0 / 839) | | **2.3**  (19 / 840) | | **4.4** (37 / 840) | | **18.8** (308 / 1637) | | | |

**“Remote” notifications and swabs. The “%” figures are the contribution that each cell makes to the total for that column.**

|  | Remote | | | |
| --- | --- | --- | --- | --- |
|  | East Arnhem | | Central Australia | |
|  | Notifications (%) | Swabs (%) | Notifications (%) | Swab (%) |
| *C. trachomatis* | 193 (25.1) | 14 (12.2) | 377 (29.0) | 23 (10.8) |
| *N. gonorrhoeae* | 80 (10.4) | 3 (2.6) | 598 (46.1) | 86 (40.4) |
| *T. vaginalis* | 496 (64.5) | 98 (85.2) | 323 (24.9) | 104 (48.8) |

**“Regional” notifications and swabs**

|  | Regional | | | |
| --- | --- | --- | --- | --- |
|  | East Arnhem | | Barkly | |
|  | Notifications (%) | Swabs (%) | Notifications (%) | Swabs (%) |
| *C. trachomatis* | 193 (25.1) | 0 (0) | 79 (30.7) | 7 (41.2) |
| *N. gonorrhoeae* | 80 (10.4) | 0 (0) | 64 (24.9) | 0 (0) |
| *T. vaginalis* | 496 (64.5) | 20 (100) | 114 (44.4) | 10 (58.8) |

**“Sexual Health Clinic” notifications and swabs**

|  | Sexual Health Clinic | |
| --- | --- | --- |
|  | Notifications (%) | Swabs (%) |
| *C. trachomatis* | 400 (79.2) | 0 (0) |
| *N. gonorrhoeae* | 71 (14.1) | 9 (90) |
| *T. vaginalis* | 34 (6.7) | 1 (10) |
